# Respiratory function in 192 adult patients with spinal muscular atrophy (SMA) treated with nusinersen – a multicenter observational study

**DOI:** 10.1186/s13023-025-04009-3

**Published:** 2025-09-08

**Authors:** Claudia D. Wurster, Benjamin Stolte, Tobias Kessler, Maren Freigang, Bogdan Bjelica, Benjamin Ilse, Jan C. Koch, Isabell Cordts, Alexander Mensch, Daniel Zeller, Zeljko Uzelac, Georges Sam, Hanna Sophie Lapp, Camilla Wohnrade, Annekathrin Rödiger, Mohamad Tareq Muhandes, Ilka Schneider, Julia Bellut, Julia Nentwich, Johannes Dorst, Joachim Schuster, Olivia Schreiber-Katz, Alma Osmanovic, Andreas Totzeck, Andreas Thimm, Robert Steinbach, Julian Grosskreutz, Christoph Kleinschnitz, Albert C. Ludolph, Marcus Deschauer, Janbernd Kirschner, Jens Dreyhaupt, Kurt Wollinsky, Susanne Petri, Markus Weiler, Tim Hagenacker, René Günther

**Affiliations:** 1 Institute of Human Genetics, Ulm University and Ulm University Medical Center, Albert-Einstein-Allee 11, 89081 Ulm, Germany; 2 Department of Neurology, Ulm University Medical Center, Ulm, Germany; 3 Department of Neurology and Center for Translational Neuro- and Behavioral Sciences (C-TNBS), University Hospital Essen, Essen, Germany; 4 Department of Neurology, Heidelberg University Hospital, Heidelberg, Germany; 5 Clinical Cooperation Unit Neurooncology, German Cancer Consortium (DKTK), German Cancer Research Center (DKFZ), Heidelberg, Germany; 6 Department of Neurology, University Hospital Carl Gustav Carus at Technische Universität Dresden, Dresden, Germany; 7 Department of Neurology, Hannover Medical School, Hannover, Germany; 8 Department of Neurology, Jena University Hospital, Jena, Germany; 9 Department of Neurology, University Medicine Göttingen, Göttingen, Germany; 10 Department of Neurology, Klinikum rechts der Isar, School of Medicine and Health, Technical University of Munich, Munich, Germany; 11 Department of Neurology, University Medicine Halle, Halle (Saale), Germany; 12 Department of Neurology, University Hospital Würzburg, Würzburg, Germany; 13 Center for Rare Diseases, Jena University Hospital, Jena, Germany; 14 Department of Neurology, St. Georg Hospital Leipzig, Leipzig, Germany; 15 German Center for Neurodegenerative Diseases, DZNE, Site Ulm, Ulm, Germany; 16 Essen Centre for Rare Diseases (EZSE), University Hospital Essen, Essen, Germany; 17 Precision Neurology of Neuromuscular and Motor Neuron Diseases, University of Lübeck, Lübeck, Germany; 18 Cluster for Precision Medicine in Chronic Inflammation, Universities of Kiel and Lübeck, Lübeck, Germany; 19 Department of Neuropediatrics and Muscle Disorders, Faculty of Medicine, Medical Center - University of Freiburg, University of Freiburg, Freiburg, Germany; 20 Institute of Epidemiology and Medical Biometry, Ulm University, Ulm, Germany; 21 Department of Anesthesiology, RKU, Ulm University, Ulm, Germany; 22 Deutsches Zentrum für Neurodegenerative Erkrankungen (DZNE), Dresden, Germany

**Keywords:** Spinal muscular atrophy (SMA), Nusinersen, Respiratory function, Forced vital capacity (FVC), Adult patients

## Abstract

**Background:**

Natural history data show that respiratory function is impaired in SMA patients. Observational studies have shown stabilization of respiratory function in adult SMA patients treated with nusinersen. However, long-term studies investigating the effect of nusinersen on respiratory function in adult SMA patients are rare.

**Methods:**

We examined respiratory function using forced vital capacity of predicted normal (FVC%), FVC in liters, capacity per second (FEV1) and peak expiratory flow (PEF) in 192 adult SMA patients treated with nusinersen for a median of 3.2 years (IQR: 2.1–4.0, range: 0.2–5.2). Changes in spirometric parameters were analyzed using individual linear regression models separate in each patient to estimate the slope. Additionally, three multivariable models were performed to assess the effect of age, sex, treatment duration, baseline FVC% and each one of the variables of interest (1) SMA type, (2) ambulation status, (3) spondylodesis on follow-up FVC%. Associations between respiratory parameters and motor function (HFMSE) were investigated via Scatter plots and Spearman’s rank correlation.

**Results:**

Spirometric parameters remained stable during treatment (median annual rate of change of FVC% 0.17% (*p* = 0.40), FVC in liters  -0.002 (*p* = 0.59), FEV1 -0.014 l (*p* = 0.06) and PEF 0.025 l/s (*p* = 0.65)). In all multivariable models, age, sex, treatment duration, SMA type, ambulation status, and spondylodesis showed no significant association with follow-up FVC%. No significant correlations were observed between respiratory and motor function.

**Conclusion:**

Respiratory parameters remained stable during treatment with nusinersen in adult SMA patients over several years.

**Clinical trial number:**

Not applicable.

## Background

Spinal muscular atrophy (SMA) is a neuromuscular disease caused by a homozygous loss of the survival of motoneuron- (*SMN1*) gene function [1, 2]. The lack of SMN protein leads to a degeneration of alpha motor neurons in the spinal cord and brain stem and thus to muscle weakness and muscle atrophy. Small amounts of SMN protein are synthesized via a highly homologous gene, the *SMN2* gene [3]. *SMN2* copy number is the most important determinant of phenotype, and a high copy number usually leads to a milder course of disease [4]. Even though a new classification based on the patient’s current motor skills (e.g. non-sitter, sitter, walker) is now used [5, 6], the division into different types (0–4, from severe to mild) depending on the age of disease onset and the achievement of motor milestones [7] is still common.

Patients with severe SMN protein deficiency develop respiratory insufficiency due to the involvement of respiratory muscles, which in the past was usually fatal, especially for SMA type 1, but also for SMA type 2 and occasionally SMA type 3 patients [8, 9]. Typically, the intercostal muscles are more affected than the diaphragm [10–12], which results in a paradoxical breathing pattern and a bell-shaped chest in severely affected patients [13, 14]. Scoliosis and joint contractures, which often manifest in the natural course of the disease, lead to further deformation and constriction of thorax and lungs and aggravate the ventilation disorder. Secretion retention and aspiration frequently cause respiratory infections, which in turn can lead to dys- and atelectasis and thus further increase the restriction [15].

Since respiratory function in the acute, infantile form (SMA type 1) already deteriorates massively at a very early stage in life in the natural course of disease, surveys of spirometric parameters such as forced vital capacity (FVC%) are usually not available for this patient group and are limited to the chronic forms of disease progression (SMA types 1c to 4). Previous studies show that FVC% decline in untreated SMA type 2 and 3 patients is most pronounced at younger ages, followed by a slower rate of decline or stabilisation in adolescence and adulthood [16, 17]. Average annual FVC% decline rates differed significantly between SMA types and ages, ranging from − 0.2% to -1.4% (related to an SMA collective aged 4 to 74 years) [17].

The antisense-oligonucleotide (ASO) nusinersen was the first disease-modifying drug approved for the treatment of SMA. Evidence for the efficacy of nusinersen on motor function has been demonstrated for both children [18, 19] and adults with SMA [20–22]. Real-world studies investigating treatment effects of nusinersen on respiratory function in children with SMA ranged between improvement to stabilization to slower decline of respiratory function [23–26]. In adult SMA patients, one study showed an improvement in diaphragmatic motility in adult SMA patients treated with nusinersen [12], while the spirometric parameters were described as essentially stable [12, 27–32]. However, these studies are limited and often include only small numbers of cases in observation periods up to 22 months (median) [27–34].

A recent systematic review analyzed 13 studies involving 646 SMA patients across different age groups and clinical types. The review found a general trend toward improved respiratory function, particularly FVC%, with additional positive trends in peak expiratory flow (PEF) and peak cough flow (PCF), although not all findings were statistically significant. Importantly, the most pronounced improvements were observed in younger patients and those who initiated nusinersen therapy early. In contrast, effects in older or more advanced cases were less consistent [35].

In this study, we evaluated the long-term effects of nusinersen on respiratory function in the largest cohort of adult SMA patients to date, with a follow-up period of up to five years. This work extends the findings from our previous single-center investigations on this topic [27, 32].

## Methods

### Study design and participants

SMA patients for this observational, multicenter study were enrolled between July 2017 and December 2022 at the Departments of Neurology in Dresden, Essen, Göttingen, Halle, Hannover, Heidelberg, Jena, Munich, Ulm and Würzburg (in total 10 sites in Germany).

Inclusion criteria were genetically-confirmed, 5q-associated SMA due to homozygous deletion of exons 7, 8, or both, or to compound heterozygous *SMN1* mutations, nusinersen treatment administered according to the official prescribing information, age ≥ 16, baseline and ≥ 1 follow-up respiratory measurement available. Exclusion criteria were spinal disease and/or coagulation disorder (or inability to discontinue anticoagulant medication) that would preclude intrathecal nusinersen delivery, limited mouth/jaw opening that would preclude spirometry and lack of informed written consent to participate in the study. Patients’ history and clinical data were collected before therapy started (s. Table 1). SMA type classification was based on the onset of disease and motor skills achieved. Patients received intrathecal administrations of 12 mg nusinersen following the standard of care dosing schedule (treatment day 0, 14, 28 and 63 and every 4 months after that). Intrathecal injection of nusinersen was performed by standard access. For patients with severe scoliosis a CT- or fluoroscopy-guided lumbar puncture was carried out [36]. All patients were treated according to the current standard of care [5, 6].

### Spirometry

Spirometry was performed along with the intrathecal administration of nusinersen on treatment days 0 (baseline, T1), 63 (T4) and every four months (visit T5 to T19). Spirometry was performed by an experienced examiner and in accordance with national guidelines [37, 38]. Depending on availability in the respective centers, appropriate spirometry devices were used (in the Department of Neurology at Ulm University Hospital e.g. a hand manometer (ProSpiro mobile edition, MESA Medizintechnik GmbH, Benediktbeuren, Germany)). Respiratory parameters were measured either in sitting or in supine position [17]. Usually a mouthpiece was used to carry out spirometry. Patients performed at least three breathing maneuvers, with the best attempt scored. FVC, as the most meaningful spirometry parameter for restrictive ventilation disorders, is defined as the maximum volume exhaled as quickly as possible after complete inhalation under the greatest possible exertion. FVC was measured in liters and indicated in litres (FVC (l)) and in % predicted of individual normal (FVC%) as spirometric values are influenced by different factors (e.g. age, sex, and height; see reference values published by the GLI (Global Lung Function Initiative)) [39]. In addition to FVC, forced expiratory volume in one second (FEV1) and PEF were recorded. FEV1, given in liters (l) and defined as the forced expiratory volume that can be exhaled within the first second (capacity per second), is a parameter that can help to identify an obstructive component of a ventilation disorder. PEF, given in liters per second (l/s), indicates the maximum respiratory flow rate during exhalation and is often equated with the coughing burst.

### HFMSE

The Hammersmith Functional Motor Scale Expanded (HFMSE) was used to evaluate the relationship between changes in respiratory parameters and motor function during nusinersen therapy. The HFMSE comprises 33 items designed to assess global motor performance in patients with SMA. Each item is scored on a 3-point scale (0 to 2), with higher scores indicating better motor function, resulting in a maximum total score of 66 points [40, 41].

### Statistics

Continuous data were described by median, quartiles (IQR) and range; categorical data as frequencies (percentage (%)). To investigate the rate of changes over time in FVC%, FVC (l), FEV1 and PEF, individual linear regression models were used separate in each patient to estimate the slope (individual patient changes per year). The Wilcoxon signed rank test was used for further investigations of these slopes.

To evaluate factors associated with respiratory function during nusinersen treatment, we conducted three separate multivariable linear mixed-effects models. Each model included age, sex, treatment duration and baseline FVC% as covariates. In addition, each model incorporated one clinical variable of interest to investigate subgroup effects: SMA type, ambulation status, or history of spinal fusion surgery (spondylodesis). The regression coefficients incl. 95% confidence interval and *p*-values are shown as main results from linear mixed effects regression analysis.

Associations between respiratory parameters (FVC%, FEV1 and PEF) and motor function measured by HFMSE were investigated by scatter plots and Spearman’s rank correlation coefficient. Because of the explorative character of this study, the results of the statistical tests were not interpreted as confirmatory but as hypothesis generating only. Accordingly, no adjustment for multiple testing was done. A two-sided *p*-value < 0.05 was interpreted as statistically significant. Statistical analysis was performed with SAS 9.4 under Windows. Figures were created using R, version 4.3.2.

## Results

286 SMA patients were screened for the study. Considering inclusion and exclusion criteria, 192 patients were finally eligible for the study. Of these, 44% were women and 56% men. Median age was 34.3 years (IQR: 25.8–48.3, range: 16.0–68.2). Two patients were classified as SMA type 1, 60 as SMA type 2, 125 as SMA type 3 and 5 as SMA type 4. 30% of the SMA patients in our cohort were walkers. Two thirds of the SMA patients (67%) had scoliosis and 22% of the patients have had spondylodesis (spinal fusion) surgery in the past. Median HFMSE of the entire SMA cohort was 10.0 (IQR: 2.0–39.5, range: 0.0–66.0) at baseline.

Median FVC% of the entire SMA cohort was 75.0% (IQR: 36.2–90.4, range: 7.9–126.8) at the start of Nusinersen therapy. Stratified by subgroups, the two SMA type 1 patients had a FVC% of 10.5% respectively 7.9%. The SMA type 2 cases showed a median FVC% of 24.5% (IQR: 17.4–44.1, range: 8.5–78.8). SMA type 3 patients (non-ambulatory and ambulatory SMA patients) had a median FVC% of 84.3% (IQR: 71.2–95.9, range: 9.8–126.8) with 80.0% (IQR: 61.5–88.6, range: 9.8.–126.8) in the non-ambulant SMA type 3 group and 91.7% (IQR: 82.4–99.8, range: 57.9–117.6) in the ambulant SMA type 3 group. The SMA type 4 patients showed a median FVC% of 95.6% (IQR: 86.0–111.4, range: 82.9–121.2).

Median values of FVC in litres, FEV1 and PEF of the entire SMA cohort are shown in Table 1.

More than half of the adult SMA patients (57%) had impaired lung function (FVC% ≤ 80%) at baseline. Regarding the SMA subtypes, all of the SMA type 1 and SMA type 2 patients had a restricted FVC% ≤ 80% at baseline, compared to 38% of SMA type 3 and none of the SMA type 4 patients. Furthermore, 25% had a non-invasive ventilation (NIV). Of the SMA patients who used NIV, two thirds were SMA type 1 and 2 patients (69%) and one third were SMA type 3 patients (31%). None of the ambulatory SMA type 3 patients and none oft the SMA type 4 patients used NIV.

Detailed baseline characteristics of all SMA patients are shown in Table 1.

**Table 1:** Demographics and baseline functional assessment. Categorical data are described as frequencies (percentage (%)); continuous data by median, quartiles (IQR) and range (in brackets)

SMA features	
*N*	192 (100%)
SMA type (*n*, %) SMA type 1 SMA type 2 SMA type 3 SMA type 4	2 (1%) 60 (31%) 125 (65%) 5 (3%)
Male gender (*n*, %)	107 (56%)
Age at start therapy (years)	34.3 (IQR: 25.8–48.3, range: 16.0–68.2)
Ambulatory Wheelchair In-between	58 (30%) 119 (62%) 15 (8%)
BMI	22.8 (IQR 19.5–27.4, range 8.1–52.2)
PEG/NG tube	7 (4%)
Scoliosis Spondylodesis	129 (67%) 43 (22%) *(N/A: 18)*
NIV	48 (25%)
FVC% baseline FVC (l) baseline FEV1 (l) baseline PEF (l/s) baseline	75.0 (IQR: 36.2–90.4, range: 7.9–126.8) 2.9 (IQR: 1.3–4.1, range: 0.2–6.9) 2.5 (IQR: 1.1–3.5, range: 0.2–5.8) 4.7 (IQR: 2.4–7.3, range: 0.4–12.3)
HFMSE baseline	10.0 (IQR: 2.0–39.5, range: 0.0–66.0)
*SMN2* copy number (*n*, %) < 4 ≥ 4	98 (51%) 77 (40%) *(N/A: 17)*

Abbreviations: SMA = spinal muscular atrophy, BMI = Body Mass Index, PEG = percutaneous endoscopic gastrostomy, NG tube = nasogastric tube, NIV = Non-invasive ventilation, FVC = forced vital capacity, FEV1 = forced expiratory volume in one second, PEF = peak expiratory flow, HFMSE = Hammersmith Functional Motor Score Expanded, *SMN*2 = survival of motoneuron 2 (gene), N/A: not available

Patients were observed for a median time of 3.2 years (IQR: 2.1–4.0, range: 0.2–5.2).

**Fig. 1:**
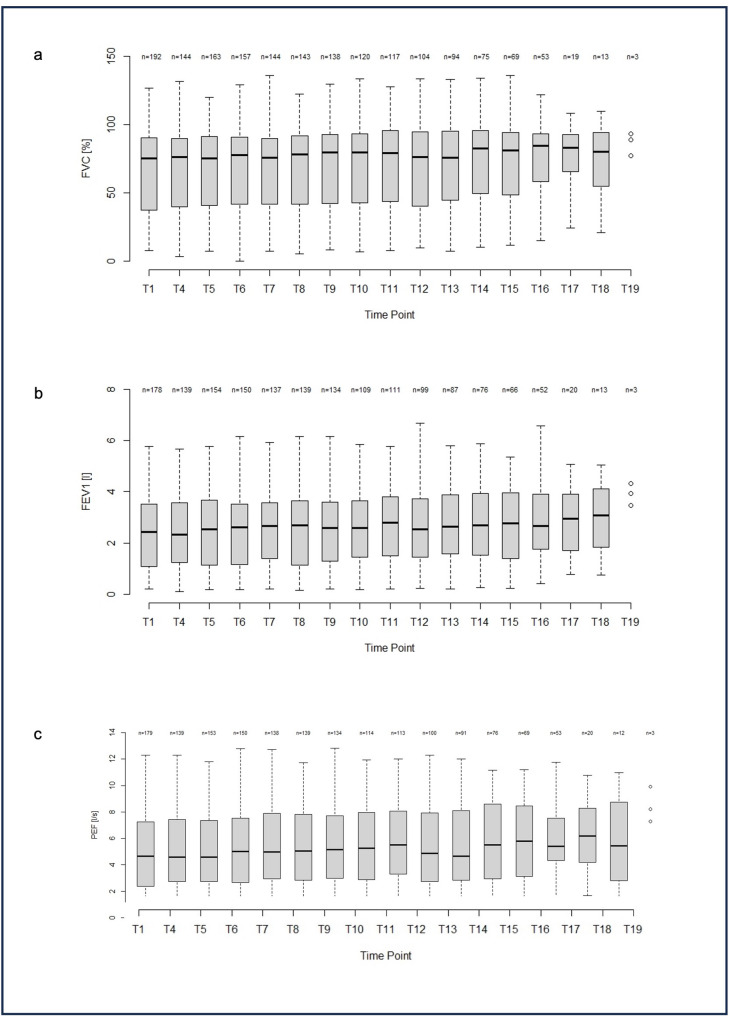
**a**: Boxplot FVC% of the entire SMA cohort. Boxplots of FVC% at T1 (baseline, start of treatment with nusinersen) to visit T19. Boxplots show the median and IQR, whiskers show the range. A dot plot was used for visit T19 due to the low number of observations. **b**: Boxplot FEV1 (l) of the entire SMA cohort. Boxplots of FEV1 (l) at T1 (baseline, start of treatment with nusinersen) to visit T19. Boxplots show the median and IQR, whiskers show the range. A dot plot was used for visit T19 due to the low number of observations. **c**: Boxplot PEF (l/s) of the entire SMA cohort. Boxplots of PEF (l/s) at T1 (baseline, start of treatment with nusinersen) to visit T19. Boxplots show the median and IQR, whiskers show the range. A dot plot was used for visit T19 due to the low number of observations

Spirometric paramters of the entire study population did not change during treatment. The median rate of change in FVC% for all SMA patients over time was 0.17% per annum (IQR: -1.42–1.62, range: -31.69–37.76) (*p* = 0.40, Fig. 1a). The annual rate of median change in FVC in litres for all SMA patients over time was  -0.002 (IQR: -0.065–0.055, range: -1.008–1.086) (*p* = 0.59, not shown). FEV1 (in l) of the entire SMA group showed a median rate of change of -0.014 per annum (IQR: -0.073–0.043, range: -0.667–0.813) (*p* = 0.06, Fig. 1b) and PEF (in l/s) a median rate of change of 0.025 per annum (IQR: -0.153–0.126, range: -4.445–3.865) (*p* = 0.65, Fig. 1c).

The three multivariable linear mixed-effects models evaluating factors associated with respiratory function during nusinersen treatment consistently identified baseline FVC% as the strongest and only statistically significant predictor of follow-up FVC% across all models (all *p* < 0.0001).

In the model including SMA type, neither SMA type, treatment duration, age, nor sex showed a statistically significant association with follow-up FVC%.

In the model assessing ambulation status, walkers exhibited significantly higher FVC% values than those with permanent wheelchair dependency (*p* = 0.047). But again, neither ambulation status nor treatment duration, age, or sex showed a statistically significant association with follow-up FVC%.

In the third model, spondylodesis was not significantly associated with follow-up FVC%, and the results for age, sex, and treatment duration remained non-significant.

The results from the multivariable linear mixed-effects models are shown in Table 2.

**Table 2:** Estimated treatment effects on FVC% from multivariable models during Nusinersen therapy. Regression results from multivariable linear mixed-effects models including baseline FVC%, age, sex and treatment duration as covariates. The table presents estimated fixed effects (regression coefficients incl. 95% confidence limits) of the main clinical variable in each model, as well as the regression coefficient for treatment duration

Clinical variable	Effect estimate	*p*-value	Rate of change (per year)	*p*-value
SMA type 1 SMA type 2 SMA type 3 vs. type 4	-7.19 (95% CI: -17.86–3.49) -3.99 (95% CI: -10.36–2.38) -2.84 (95% CI: -8.34–2.66)	0.19 0.22 0.31	0.08 (95% CI: -0.13–0.30)	0.45
ambulation status walker in-between vs. wheelchair-bound	2.33 (95% CI: 0.03–4.64) 1.11 (95% CI: -2.30–4.52)	0.047* 0.52	0.06 (95% CI: -0.15–0.27)	0.57
Spondylodesis no vs. yes	0.79 (95% CI: -1.68–3.25)	0.53	0.04 (95% CI: -0.17–0.26)	0.70

Abbreviations: SMA = spinal muscular atrophy

The scatter plots showed no recognizable association between the individual slopes of FVC%, FEV1, PEF and the slope of HFMSE (not shown). The correlation coefficient according to Spearman was 0.10 (*p* = 0.19) for FVC% and HFMSE, -0.01 (*p* = 0.84) for FEV1 and HFMSE and  -0.12 (*p* = 0.11) for PEF and HFMSE. Furthermore, there were no recognizable association between the individual slopes of FVC% and FVC% at baseline or HFMSE at baseline as according to Spearman’s correlation coefficients of -0.05 (*p* = 0.46) and 0.02 (*p* = 0.75) respectively.

## Discussion

This multicenter observational study investigated long-term respiratory parameters in a cohort of 192 adult patients with SMA treated with nusinersen over a median period of 3.2 years. We observed stability in spirometric parameters in the overall SMA cohort during treatment with nusinersen. This aligns with findings from previous smaller studies including our previous single-center studies [27, 32] and contributes long-term real-world evidence to the limited data landscape [35].

As no control group was included in this study, conclusions regarding the treatment effect are limited. Most studies assessing the natural history of respiratory function in type 2 and 3 SMA patients have focused primarily on pediatric populations [16, 42]. Available studies indicate a non-linear pattern of FVC decline in (types 1c to 3a) SMA, with the most significant reductions typically occurring in childhood and early adolescence, followed by a slower rate of decline or even stabilization in adulthood [16, 17, 43]. In SMA type 3b, FVC tends to remain relatively stable across the lifespan [17]. Despite these insights, the natural course of respiratory function in adult SMA patients remains insufficiently characterized, as adult-specific longitudinal data are scarce.

Age, sex, and treatment duration did not show a significant association with changes in FVC%. Likewise, SMA type had no statistically relevant impact on how FVC% evolved over time under therapy. This suggests that disease severity as defined by SMA subtype did not meaningfully affect the respiratory response to nusinersen. Ambulation status, however, was associated with absolute FVC% values: ambulatory patients had significantly higher FVC% compared to those who were permanently wheelchair-dependent. However, this difference was limited to the level of FVC% and did not reflect a different pattern of change over time. Similarly, a history of spinal fusion surgery (spondylodesis), which could potentially influence thoracic mechanics and breathing capacity, was not significantly associated with FVC% outcomes.

There was no correlation between individual changes in spirometric parameters (FVC%, FEV1, PEF) and changes in motor function as measured by the HFMSE. This lack of association may reflect the overall stability of respiratory parameters in our cohort. However, it is also possible that the responsiveness of the HFMSE in adult patients is limited. Previous studies have described both floor effects in patients with low motor function and ceiling effects in especially ambulatory individuals, which may reduce sensitivity to change in these subgroups [41].

While spirometry is a standard method to assess respiratory function, it may not fully capture all aspects of respiratory involvement in SMA. Future studies could consider including additional outcome measures such as peak cough flow, diaphragmatic ultrasound, or capnography to further characterize respiratory changes in this population. Nevertheless, studies that map the long-term course of spirometric parameters in treated adult SMA patients even beyond the period covered by our study are still necessary.

## Conclusions

Respiratory parameters remained stable during treatment with nusinersen in adult SMA patients over several years.

## Data Availability

The datasets used and/or analysed during the current study are available from the corresponding author on reasonable request.
